# Aortic valve replacement in octogenarians: performance analysis of EuroSCORE II

**DOI:** 10.1186/1749-8090-8-S1-O102

**Published:** 2013-09-11

**Authors:** D Hernandez-Vaquero, R Diaz, JC Llosa

**Affiliations:** 1 Cardiac Surgery Department, Central University Hospital of Asturias, Oviedo, Spain

## Background

EuroSCORE has been proposed to identify patients at high risk for surgical aortic valve replacement and estimate for them the risk-benefit of percutaneous implantation. However, it has been clearly demonstrated that this system overestimates mortality, particularly in the elderly population undergoing surgery for aortic valve replacement. An actualized model called EuroSCORE II has been recently proposed to predict mortality after cardiac surgery but it has not been validated yet in the elderly population undergoing aortic valve replacement. We aimed to validate the new risk model in an octogenarian population who underwent surgery for aortic valve replacement in our center.

## Methods

From March 2005 to March 2013, 378 consecutive octogenarian patients underwent aortic valve replacement in our center. Calibration (Hosmer-Lemeshow test and risk-adjusted mortality ratio) and discrimination (area under the receiver-operating characteristic curve) were calculated to assess the capacity of EuroSCORE II to predict 30-day mortality. Moreover, the same analysis was made in a subgroup of patients who underwent isolated aortic valve replacement.

## Results

Observed mortality was 8,7% in the overall population and the predicted mortality by EuroSCORE II was 6,31% so that it underestimated the real mortality rate. However, the general calibration assessed by Hosmer-Lemeshow test was not bad (p=0,15) and the discriminatory power was very good (C-Statistic= 0,90). Assessing calibration by quartiles of risk, an adequate predictive power was observed in the first three quartiles but not in the fourth (Hosmer-Lemeshow, p=0,032; risk adjusted mortality ratio=1,93). Similar results were obtained in the subgroup of patients who underwent isolated replacement.

## Conclusions

EuroSCORE II is a good model for predicting mortality after surgery for aortic valve replacement in octogenarians. However, this system underestimates the mortality rate in the highest quartile of risk.

